# Anti-Inflammatory Properties of the Medicinal Mushroom *Cordyceps militaris* Might Be Related to Its Linear (1→3)-β-D-Glucan

**DOI:** 10.1371/journal.pone.0110266

**Published:** 2014-10-17

**Authors:** Fhernanda R. Smiderle, Cristiane H. Baggio, Débora G. Borato, Arquimedes P. Santana-Filho, Guilherme L. Sassaki, Marcello Iacomini, Leo J. L. D. Van Griensven

**Affiliations:** 1 Department of Biochemistry and Molecular Biology, Federal University of Parana, Curitiba, Parana, Brazil; 2 Department of Pharmacology, Federal University of Parana, Curitiba, Parana, Brazil; 3 Plant Research International, Wageningen University and Research Centre, Wageningen, The Netherlands; Shanghai University of Traditional Chinese Medicine, China

## Abstract

The Ascomycete *Cordyceps militaris*, an entomopathogenic fungus, is one of the most important traditional Chinese medicines. Studies related to its pharmacological properties suggest that this mushroom can exert interesting biological activities. Aqueous (CW and HW) and alkaline (K5) extracts containing polysaccharides were prepared from this mushroom, and a β-D-glucan was purified. This polymer was analysed by GC-MS and NMR spectrometry, showing a linear chain composed of β-D-Glc*p* (1→3)-linked. The six main signals in the ^13^C-NMR spectrum were assigned by comparison to reported data. The aqueous (CW, HW) extracts stimulated the expression of IL-1β, TNF-α, and COX-2 by THP-1 macrophages, while the alkaline (K5) extract did not show any effect. However, when the extracts were added to the cells in the presence of LPS, K5 showed the highest inhibition of the pro-inflammatory genes expression. This inhibitory effect was also observed for the purified β-(1→3)-D-glucan, that seems to be the most potent anti-inflammatory compound present in the polysaccharide extracts of *C. militaris. In vivo*, β-(1→3)-D-glucan also inhibited significantly the inflammatory phase of formalin-induced nociceptive response, and, in addition, it reduced the migration of total leukocytes but not the neutrophils induced by LPS. In conclusion, this study clearly demonstrates the anti-inflammatory effect of β-(1→3)-D-glucan.

## Introduction

The scientific community have provided plenty of data showing that mushroom extracts demonstrate interesting biological properties such as antitumor [1], anti-inflammatory [2], antiviral [3], and immunomodulatory effects [4], [5], [6], [7]. These extracts may contain different molecules as steroids, polyphenols, hydroquinones, triterpenes, proteins, glycoproteins, and polysaccharides that are involved in such biological effects [3].

Several mushrooms have been studied for their pharmacological potentials. Among them, *Cordyceps militaris*, an entomopathogenic fungus belonging to the class Ascomycetes, is the one of the most important traditional Chinese medicines, being the second most commercialized medicinal mushroom species in China, Korea, and Japan [8], [9]. *C. militaris* is used as a folk tonic in East Asia and the studies related to its pharmacological properties suggest that this mushroom can exert antioxidant, antiviral, hypoglycemic, and immuno-protective activities [9], [10], [11], [12]. Some of these effects are attributed to the polysaccharides, as β-glucans, although other active components such as cordycepin, ergosterol, and mannitol may also be responsible for an increase in the ATP production and in the oxygen utilization [13].

The glucans isolated from mushrooms (Basidiomycetes and Ascomycetes) up to date include α-glucans, being the most common branched (1→4),(1→6)-α-D-glucans; and β-glucans, which can be linear (1→3)- or (1→6)-linked, or branched (1→3),(1→6)-β-D-glucans [14]. Many of fungal glucans exhibit biological activity and they belong to a group of physiologically active compounds that are named biological response modifiers (BRMs). The glucans presenting β-configuration have shown to be the most effective BRMs, and their activity may vary according to their molecular weight, degree of branching and conformation in solution, although these data require further investigation [14], [15], [16].

The possible mechanism of action of such molecules is based on the recognition of β-glucans by cell surface receptors called pattern recognition receptors (PRRs). The PRRs recognize the β-glucans as pathogen-associated molecular patterns (PAMPs) and initiate immune responses. In humans, a number of such receptors have been identified, as dectin-1, complement receptor 3 (CR3), scavenger receptors, lactosylceramide (LacCer), and toll-like receptors (TLRs) [15], [16].

Among the immune responses initiated by these polymers are the activation of leucocytes, stimulation of phagocytic and cytotoxic activities, and production of pro-inflammatory mediators by cells of the immune system [17]. The antitumor and immune-stimulating activities are the most studied effects, although some mushroom extracts have presented anti-inflammatory properties [2], [18], [19].

Inflammation is a beneficial host response to infection and to tissue injury that ultimately leads to the restoration of normal tissue structure and function. A normal inflammatory response is self-limiting, although prolonged inflammation contributes to pathogenesis of many inflammatory diseases [2], [20] and to cancer [21], [22].

Some authors demonstrated that 70% ethanolic extracts from *Cordyceps militaris* showed topical anti-inflammatory activity in the croton oil-induced ear edema in mice [23]. The hot water extract from the same mushroom have also presented anti-inflammatory effect *in vitro*, by the reduction of LPS-induced production of NO, TNF-α, and IL-6 secretion by RAW 264.7 cells [2].

The mushroom *Cordyceps militaris* is vastly appreciated for its medicinal properties, although little is known about the effect of its polysaccharides. Therefore, this study aims to isolate and characterize its β-glucan and evaluate the anti-inflammatory activities of its polysaccharide extracts and the purified β-glucan.

## Experimental

### Fungal material

Fruiting bodies of *Cordyceps militaris* (L.) Link (strain: MCI 10304, Mushtech Cordyceps Institute) were a kind gift from Dr. J. M. Sung of Kangwon National University (Chuncheon, Korea).

### Isolation of the β-D-glucan

**Figure 1:**
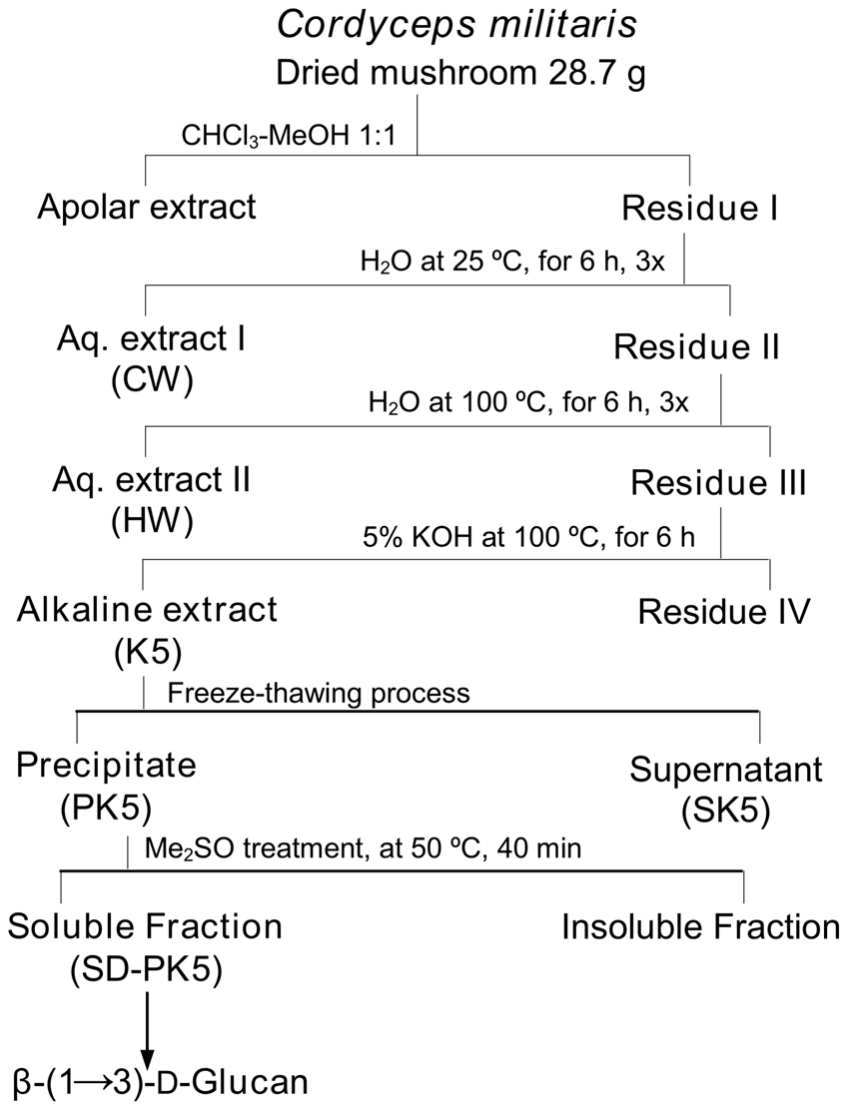
Extraction and purification steps of the β-D-glucan (SD-PK5) of *C. militaris*.

The dried mushroom (28.7 g) was submitted to several extraction steps as shown in figure 1. Briefly, the material was firstly treated with CHCl_3_:MeOH (1∶1, v/v), using a Soxhlet, under heating (50°C), for 3 days. After removing the apolar compounds and the excess of solvents, the residue I was successively submitted to cold (25°C) and hot (100°C) aqueous extractions (for 6 h, 3x for each extraction). The aqueous extracts were kept for the experiments on THP-1. The remaining residue III was extracted twice with 5% KOH solution at 100°C, for 6 h, giving rise to an alkaline extract (K5), which was neutralized with glacial acetic acid and dialysed (12–14 kDa), for 24 h. The extract (K5) was solubilized in water and submitted to freezing followed by mild thawing at 4°C [24]. This process was repeated 5 x to guarantee a complete separation of the water-soluble (SK5) of the non-soluble (PK5) polysaccharides. Both fractions were separated by centrifugation (12,000 rpm, at 4°C, for 20 min), and freeze-dried. The insoluble fraction (PK5) was the focus of this study, after a treatment with Me_2_SO (80 mL), for 40 min, at 50°C. The Me_2_SO-soluble material (SD-PK5) was recovered by centrifugation (10,000 rpm, at 20°C, for 15 min), dialysed against tap water, for 24 h, to remove the solvent, and freeze-dried.

### Alditol acetates preparation for monosaccharide composition analysis

Alditol acetates were prepared according to Sassaki et al. (2008) [25]. The resulting derivatives were analyzed by gas chromatography-mass spectrometry (GC-MS) using a Varian Saturn 2000R−3800 gas chromatograph coupled to a Varian Ion-Trap 2000R mass spectrometer with He as the carrier gas. A DB-225 capillary column (30 m×0.25 mm i.d.), which was maintained at 50°C during injection and then programmed to increase to 220°C at a rate of 40°C min^−1^, was used for the quantitative analysis of the alditol acetates. The products were identified by their typical retention times and electron impact profiles [26].

### Methylation analysis

Per-*O*-methylation of the isolated β-D-glucan (5 mg) was firstly carried out according to Haworth (1915) [27]. After 24 h under stirring, the sample was neutralized with glacial acetic acid and dialysed. To guarantee the total methylation, the glucan was submitted to another per-*O*-methylation using the modified method of Ciucanu & Kerek (1984) [28]. It was completely solubilized in dimethylsulfoxide (Me_2_SO, 1 mL), followed by the addition of iodomethane (MeI, 1 mL) and powdered NaOH (20 mg) [28]. The mixture was stirred for 30 min or until it turned to solid phase, which was left to react for 14–18 h. The material was re-solubilized in water, on ice, and neutralized with glacial acetic acid. After dialysis (8 kDa) against tap water to remove the excess of salts, the partially *O*-methylated polysaccharides were freeze-dried and the methylation procedure was repeated to guarantee a complete methylation of the free hydroxyls. After this step, the sample was partitioned between chloroform (3 mL) and distilled water (3 mL). The chloroform phase, containing the per-*O*-methylated derivatives, was evaporated and submitted to hydrolysis. The aqueous phase, containing salts and residues of the reaction was discarded. The per-*O*-methylated derivatives were hydrolyzed with 45% aqueous formic acid (1 mL) for 15 h at 100°C, followed by NaB^2^H_4_ reduction and acetylation as described above, to give a mixture of partially *O*-methylated alditol acetates. These derivatives were analyzed by GC-MS using the same conditions as described for alditol acetates, with the exception that the final temperature was 215°C. The derivatives were identified by the m/z of their positive ions, their typical retention times, and comparison with standards. The results were expressed as relative percentage of each component [26].

### Nuclear magnetic resonance spectroscopy

NMR spectra (^13^C- and DEPT-^13^C-NMR) were obtained using a 400 MHz Bruker Avance III spectrometer with a 5 mm inverse probe. The analyses were performed at 70°C in Me_2_SO-*d*_6_ (δ 39.7 for ^13^C signal), and the chemical shifts are expressed in δ ppm.

### Colorimetric determination of triple helix with Congo red

This experiment was performed according to Palacios et al. (2012) [29], with minor modifications. Briefly, the conformational structure of the β-D-glucan was established by helix-coil transition analysis. Congo red was dissolved in different concentrations (10–100 µM) in 50 mM NaOH solution, in order to optimize the concentrations of the dye. Dextran (Sigma, M*_w_* 40,200 g/mol) was used as control. The polysaccharide samples (Dextran 104 and β-D-glucan) were dissolved in 50 mM NaOH at 1 mg/mL and were added to Congo red solutions. Spectra were recorded on Epoch Microplate Spectrophotometer, in intervals of 10 nm from 400 to 640 nm.

### Detection of LPS contamination of the polysaccharide samples by GC-MS

The samples were subjected to methanolysis and prepared according to Santana-Filho et al. (2012) [30]. The LPS standard from *Escherichia coli* serotype O111:B4 was obtained from Sigma-Aldrich (St. Louis, MO, USA) and diluted in deionized water and the solution was then sonicated (two cycles of 15 min). An aliquot containing 300 µg of each sample was then collected, and processed as described above up to the acetylation step. Samples were gently evaporated under a N_2_ stream, and the residues dissolved in acetone (5 µL), concentrated down to 1 µL, and analyzed by GC–MS as described previously in details [30].

### Cell culture

The human monocytic cell line THP-1 (Rio de Janeiro Cell Bank, Rio de Janeiro, Brazil) was grown in RPMI 1640 culture medium (Sigma-Aldrich, cat. R8758) supplemented with 10% heat-treated newborn calf serum Sterile A (Gibco, cat. 161010–159) and 100 U/mL resp. 100 µg/mL penicillin/streptomycin (P/S) (Sigma-Aldrich), at 37°C in 5% CO_2_ in a humidified incubator. The medium was renewed twice a week.

### Macrophage differentiation and stimulation

The mature macrophage-like state was induced by treating THP-1 monocytes (500,000 cells/mL) for 48 h with 30 ng/mL phorbol 12-myristate 13-acetate (PMA; Sigma-Aldrich) in 24-wells polystyrene tissue culture plates (Costar) with 1 mL cell suspension in each well. The medium was then removed and replaced by fresh medium containing the polysaccharide fractions at 25, 50, and 100 µg/mL; or phosphate buffered saline (PBS; 50 µL), or lipopolysaccharide (LPS; 1 µg/mL, Sigma-Aldrich, cat. L-2880) as negative and pro-inflammatory controls, respectively. Cells were harvested at the time points 0 h, 3 h, and 6 h and kept in lysis buffer at −20°C for the next step. Time point 0 h was used to normalize the calculations. All experiments were performed with the same amount of cells (0.5×10^6^ per ml). The total RNA was isolated from the cells as follows.

### Cytotoxicity Assay

The cytotoxicity of the extracts and glucan added to the cells was verified by the MTT assay [31]. The MTT assay determines the viability of cells by the reduction of yellow soluble MTT in the metabolically active cells. Briefly, THP-1 monocytes were induced to differentiation into macrophages in 96-wells cell culture plate (1×10^5^ cells/well). THP-1 macrophages were exposed to different concentrations (10−250 µg/mL) of glucan or extracts and incubated for 24 h and 48 h at 37°C in 5% CO_2_ in a humidified incubator. The absorbance was measured at 595 nm using an Epoch Microplate Spectrophotometer. The experiment was performed in triplicate and the results were expressed relative to the negative control (non-stimulated cells).

### Gene expression kinetics by Real-Time PCR

Total RNA was isolated by using RNeasy mini kit (Qiagen, USA) with a RNase-free DNase (Qiagen) treatment for 15 min according to the manufacturer’s instructions. Complementary DNA (cDNA) was synthesized from isolated RNA (1 µg) with High Capacity RNA-to-cDNA kit (Applied Biosystems, USA). Expression levels of each gene were measured in triplicate reactions, performed with the same cDNA pool (1∶5 diluted), in the presence of the fluorescent dye (iQ SYBR Green Supermix) using an Real-Time PCR system (model StepOne Plus, Applied Biosystems, USA). The experiments were performed in a 20 µL reaction volume with specific primer pairs [32], and the conditions of real-time quantitative PCR were as follows: denaturation at 95°C for 10 min and amplification by cycling 40 times at 95°C for 15 s and 60°C for 60 s. Glyceraldehyde-3-phosphate dehydrogenase (GAPDH) and β-2-microglobulin were used as endogenous control, and GAPDH was chosen for normalisation. The PCR of all products were subjected to a melting curve analysis to verify the single amplification product. The relative messenger RNA (mRNA) expressions were presented as described in Chanput et al. (2010) [32]: the values were expressed as fold change relative to the value at time point zero, calculated as ΔΔCt [ΔΔCt = 2^∧^(Ct_GAPDH_ – Ct_Sample_)] [33]. The q-PCR analyses were performed twice on each sample (in triplicate), to evaluate the mRNA expression level of pro-inflammatory cytokine genes IL-1β and TNF-α and also the inflammation-related enzyme COX-2.

### Experimental Animals and ethical considerations

Experiments were conducted using female Swiss mice (25–35 g), provided by the Federal University of Parana colony. The animals were kept under standard laboratory conditions (12 h light/dark cycles, temperature 22±2°C) with food and water provided *ad libitum*. The animals were acclimatized to the laboratory for at least 12 h before testing and were used only once for experiments. All the experiments were performed after approval of the respective protocols by the Committee of Animal Experimentation of Federal University of Paraná (CEUA/BIO - UFPR; approval number 657). The study was conducted in accordance with the “Principles of Laboratory Animal Care” (NIH Publication 85–23, revised 1985) and with the ethical guidelines for investigations of experimental pain in conscious animals [34]. The number of animals and intensity of noxious stimuli used were the minimum necessary to demonstrate consistent effects of the drug treatments. After the experiments, the animals were sacrificed by cervical dislocation.

### Nociception induced by intraplantar injection of 2.5% formalin

The procedure used was similar to previously described [35]. The mice received 20 µL of a 2.5% formalin solution (0.92% formaldehyde, in saline) intraplantarly under the ventral surface of the right hind paw. Animals were observed from 0 to 5 min (early phase) and 15 to 30 min (late phase) and the time that they spent licking the injected paw was considered as indicative of nociception. Animals were treated with vehicle (saline plus 5% Me_2_SO, 10 mL/kg, i.p.) or β-D-glucan (SD-PK5) (3, 10 and 30 mg/kg, i.p), 30 min before the formalin injection.

### Peritonitis induced by intraperitoneal injection of LPS

Peritonitis was induced by LPS according to [36] with modifications. The mice were pre-treated with vehicle (saline plus 5% Me_2_SO, 10 mL/kg), dexamethasone (DEXA, a synthetic glucocorticoid, 0.5 mg/kg) or β-D-glucan (SD-PK5, 30 mg/kg) by i.p. route, 30 min before LPS injection (2 µg/kg, i.p.). Naive group received only sterile saline solution (0.9% NaCl, 10 mL/kg, i.p.). Four hours after the peritonitis induction, the mice were sacrificed and the peritoneal cavity was opened and washed with 1 mL of sterile saline (0.9% NaCl) containing heparin (25 IU/ml). Then, the peritoneal fluid was collected to determine the total number of leukocytes and levels of myeloperoxidase (MPO).

### Quantification of total leukocytes and levels of MPO

An aliquot of the peritoneal fluid was diluted with Türk solution (1∶20) and the total leukocyte counts were performed in a Neubauer chamber. To perform the measurement of MPO levels, samples of the peritoneal fluid were added to 80 mM potassium phosphate buffer (pH 5.4) containing 0.5% hexadecyltrimethylammonium bromide (HTAB), and centrifuged at 11,000 g for 20 min at 4°C. MPO levels of supernatants were determined in the presence of 0.017% H_2_O_2_ and 3,3′,5,5′-tetramethylbenzidine in dimethylformamide (TMB, 18.4 mM). The reaction was incubated at 37°C for 3 min, and then stopped by the addition of sodium acetate (1.46 M, pH 3.0). The absorbance was measured using a microplate reader at 620 nm and MPO levels were expressed as units of optic density (O.D.)/mL [37].

### Statistical Analysis

For the *in vitro* analysis, the results are expressed as mean ± standard deviation (S.D.) of duplicate cultures of three representative experiments. Statistical significance was determined using one-way analysis of variance (ANOVA) followed by Bonferroni's test, selected pairs. For the *in vivo* analysis, the data were expressed as mean ± standard error of mean (S.E.M.) with 8–10 animals per group. Comparisons between experimental and control groups were performed by one-way analysis of variance (ANOVA) followed by Newman Keul’s test. For both analyses p≤0.05 was considered statistically significant. The graphs were drawn and the statistical analyses were performed using GraphPad Prism version 5.01 for Windows (GraphPad Software, San Diego, CA, USA).

## Results and Discussion

### Isolation and characterization of the β-D-glucan

The steps of the β-glucan extraction are shown in figure 1. The yield of each fraction was calculated on the basis of initial weight of dried mushroom (28.7 g) (Table 1). The material was firstly treated with CHCl_3_:MeOH (1∶1, v/v), as described in experimental, and contained 3% apolar compounds and 97% residue I. The latter was used for the following extractions. The cold (CW) and hot (HW) water extracts were kept for the experiments on THP-1. The remaining residue III, after being extracted with 5% KOH solution, presented an extract of 9.8% yield (K5), which was submitted to freeze-thawing for several times (x 5), and centrifugation to separate the soluble polysaccharides from the non-soluble ones. This procedure gave rise to two fractions: the soluble one (SK5), that was studied previously and contained a glucogalactomannan [38]; and the insoluble one (PK5) that presented the higher amount of glucose (83%) in its composition (Table 1). The fraction PK5 was treated with Me_2_SO, which is able to solubilize mainly β-glucans, and after centrifugation, the Me_2_SO-soluble fraction (SD-PK5) was recovered showing predominantly glucose as monosaccharide component, suggesting the presence of a glucan polysaccharide (Table 1).

**Table 1:** Monosaccharide composition of the fractions obtained from *C. militaris*.

Fractions	Yield % a	Monosaccharides (mol%)^b^		
		Man	Gal	Glc
CW	3.5	37.5	34.9	27.6
HW	1.1	61.3	25.6	13.1
K5	9.8	26.8	15.2	58.0
PK5	3.7	11.0	6.0	83.0
SD-PK5 (Glucan)	1.8	Tr.**^c^**	Tr.**^c^**	98.0

Footnote:

a Calculated based on the initial dry mushroom weight.

b Alditol acetates obtained on successive hydrolysis, NaBH_4_ reduction, and acetylation, followed by GC-MS analysis.

In order to characterize the glycosidic linkages of the isolated glucan, methylation analysis was performed, and the major derivative encountered was 2,4,6-Me_3_-Glc*p* (99%), which confirmed that this polymer is a linear glucan (1→3)-linked.

**Figure 2:**
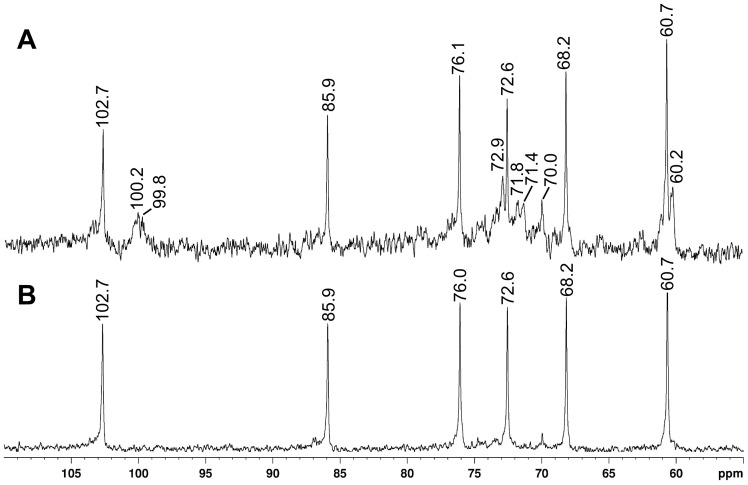
^13^C-NMR spectra of fractions PK5 (A), and SD-PK5 (B), in Me_2_SO-*d*_6_ at 70°C (chemical shifts are expressed in δ ppm).

NMR analysis ^13^C of the fractions PK5 (Figure 2A) and SD-PK5 (Figure 2B) showed that the purification procedure using Me_2_SO was efficient to remove the contaminant signals (δ 100.2; 99.8; 72.9; 71.8; 71.4; 70.0; 60.2 ppm). The six main signals in the ^13^C-NMR spectra could be attributed to the carbons of the glucose ring by comparison with reported data [14], [39], [40]. The low-field C1 signal at 102.7 ppm indicates the β-configuration, and the low-field shift of the C3 signal at 85.9 ppm indicates the 3-*O*-substitution. The non-substituted C6 was confirmed by an inverted CH_2_ signal at 60.7 ppm on DEPT experiment (data not shown). The other signals observed were attributed to C2 (δ 72.6), C4 (δ 68.2), and C5 (δ 76.0). The presence of only six signals on the SD-PK5 ^13^C-NMR spectrum is characteristic of an hexose homopolysaccharide, presenting a linear chain. These data shows that a linear β-(1→3)-D-glucan can be extracted from *C. militaris* and purified by a Me_2_SO treatment.

**Figure 3:**
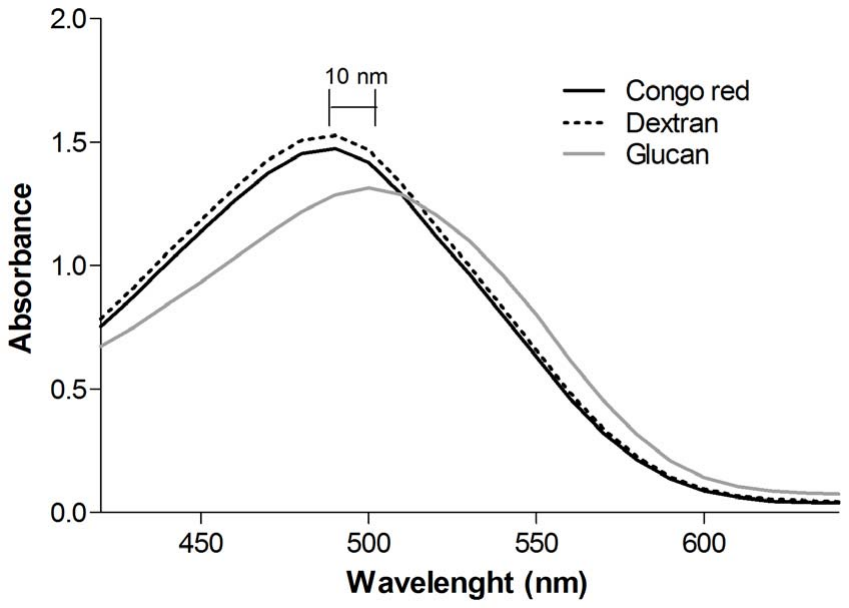
Absortion spectra of Congo Red (control), Congo Red with dextran (random coil control), and Congo Red with β-D-Glucan from *C. militaris*.

According to Ogawa et al. (1972) [41], polysaccharides existing in an ordered three-dimensional structure, generally triple helical conformation, form a complex with Congo red in dilute NaOH solutions. The complex is stabilized by strong hydrogen bonds and/or hydrophobic interactions between the polysaccharide and the dye molecule. The complex formation of polysaccharides with Congo red is commonly evaluated by means of the shift in the visible absorption maxima (λ_max_) of the Congo red spectrum [42]. The dye concentration was optimized to give a high and stable absorbance. The concentration of 80 µM gave the best result and it was chosen for the analysis [29]. The β-(1→3)-D-glucan was allowed to complex with Congo red in a 50 mM NaOH solution (Figure 3). Dextran was used as a random-coil control, and its absorbance was similar to the Congo red, which shows no complex formation. While the β-(1→3)-D-glucan sample showed a bathochromic shift (10 nm), which indicates that this polysaccharide displayed a triple helical structure.

Similar β-(1→3)-D-glucans are common in Ascomycetes and they have been isolated from *Saccharomyces cerevisiae* (curdlan) [43], and from Basidiomycete fungi as *Poria cocos* (pachyman) [14], and *Laetiporus sulphureus* [39].

### Evaluation of the anti-inflammatory properties *in vitro*

The aqueous (CW, HW) and alkaline (K5) extracts were added to the THP-1 macrophages in different concentrations (10−250 µg/mL) and tested about their cytotoxicity. None of the extracts affected the viability of the cells after 24 h or 48 h (Figure S1).

**Figure 4:**
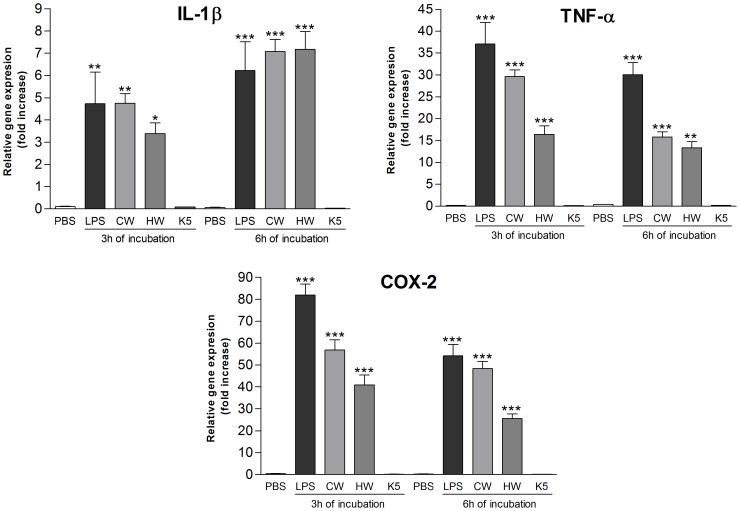
mRNA expression level of pro-inflammatory genes, after treatment with polysaccharide extracts for 3 h and 6 h. Footnote: Negative control (PBS) and positive control (LPS; 1 µg/mL). CW (cold water extract; 50 µg/mL), HW (hot water extract; 50 µg/mL), K5 (alkaline extract; 50 µg/mL). Statistical analyses were performed by means of one-way analysis of variance (ANOVA) followed by Bonferronis' test, selected pairs. The results represent the mean ± SD of duplicate cultures of three representative experiments. *p<0.05; **p<0.01; ***p<0.001 versus negative control.

Therefore, the extracts were added to the THP-1 macrophages at 50 µg/mL and the expression of pro-inflammatory genes (IL-1β, TNF-α, COX-2) was evaluated. For both incubation periods (3 h and 6 h) (Figure 4), the aqueous extracts significantly stimulated the production of IL-1β, TNF-α, and COX-2 mRNAs, while the alkaline extract did not show any stimulation, as well as the negative control (PBS).

The monosaccharide compositions of the aqueous extracts presented greater amounts of mannose and galactose, while the glucose levels were below 30%. On the other hand, the alkaline extract presented a higher amount of glucose and a lower content of the two other monosaccharides (Table 1). It was demonstrated that β-galactofuranosyl glycosides present immunomodulatory effects by the induction of phagocytic activity of macrophages and stimulation of the production of IL-1β, TNF-α, and IL-6 [44]. In our previous work [38] we isolated a glucogalactomannan from *C. militaris*, which contains 8.5% of β-galactofuranosyl in its structure. This monosaccharide may be the responsible for the stimulation of the expression of pro-inflammatory genes observed for the cells treated with CW and HW. Moreover, the galactose content of K5 is the lowest (15.2%) in comparison with the aqueous extracts, which includes both galactofuranose and galactopyranose units (Table 1). It is known that galactopyranose form does not stimulate the macrophages, therefore its presence in the alkaline extract may be the majority.

All of the extracts were tested about a possible LPS contamination, and the LPS content of CW and K5 extracts was 13.3 ng/mg and 4.0 ng/mg of dry extract, respectively. Considering that 50 µg/mL of extract was added to the cells, less than 0.7 ng/mL of LPS was present as contaminant, which is too low to interfere in the results [32]. The HW extract was negative for LPS contamination.

**Figure 5:**
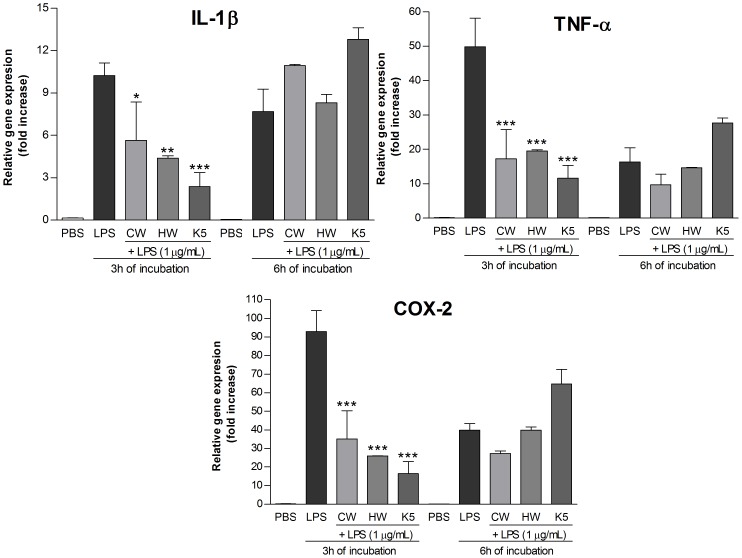
mRNA expression level of pro-inflammatory genes, after treatment with LPS+polysaccharide extracts for 3 h and 6 h. Footnote: Negative control (PBS) and positive control (LPS; 1 µg/mL). CW (cold water extract; 50 µg/mL), HW (hot water extract; 50 µg/mL), K5 (alkaline extract; 50 µg/mL). Statistical analyses were performed by means of one-way analysis of variance (ANOVA) followed by Bonferronis' test, selected pairs. The results represent the mean ± SD of duplicate cultures of three representative experiments. *p<0.05; **p<0.01; ***p<0.001 versus positive control.

In order to test the ability of the polysaccharide extracts to reduce the effects caused by LPS stimulation, 1 µg/mL of LPS plus 50 µg/mL of extracts (CW, HW, K5) were added to the cells concomitantly. After 3 h of incubation, the cells that received the extracts+LPS, showed a significant lower expression of IL-1β, TNF-α, and COX-2 mRNAs than the cells that received LPS (Figure 5). These results suggest that the *C. militaris* polysaccharide extracts exhibit an anti-inflammatory effect *in vitro*.

It is important to notice that the monosaccharide composition of CW, HW, and K5 present differences and the glucose content is the highest (58.0%) in the alkaline extract, which can be the responsible for the different stimulation of the THP-1 cells. The inhibitory effect of the LPS stimulation was more evident for the alkaline (K5) extract, inhibiting the expression of the three genes by over than 75%, as table 2 shows. The inhibition observed for CW and HW was statistically significant, although it was not stronger as for K5, which can be observed at table 2.

**Table 2:** Inhibitory effect of the extracts/glucan on the mRNA expression of pro-inflammatory genes by the THP-1 macrophages, after 3 h of incubation.

Extracts/Glucan (50 µg/mL)	Inhibitory effect on the mRNA expression (%)^a^		
	IL-1β	TNF-α	COX-2
CW	44.8±3.85	65.4±11.95	62.2±21.37
HW	57.1±0.23	60.8±0.42	72.0±0.22
K5	76.8±1.40	76.6±5.18	82.3±9.29
SD-PK5 (Glucan)	75.5±0.37	81.0±2.12	81.6±0.56

Footnote:

a Calculated based on the positive control (LPS) mRNA expression.

In a previous study, a glucogalactomannan was isolated from this mushroom and chemically characterized [38]. Although it was isolated from a soluble fraction of an alkaline extract, the aqueous extracts also showed a large amount of this heteropolysaccharide due to its high solubility in water. Nevertheless, the β-glucan, which is part of the fungal cell wall, requires a stronger method to be extracted, being present usually in higher concentrations in the alkaline extracts [45]. Therefore, we decided to proceed with an alkaline extraction, followed by a careful purification procedure with the aim to obtain high yields of the purified β-D-glucan and test its properties on the THP-1 cells.

**Figure 6:**
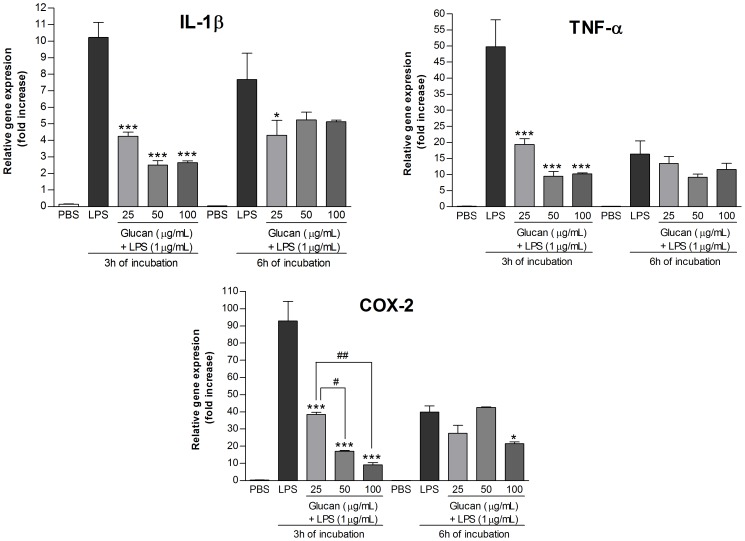
mRNA expression level of pro-inflammatory genes, after treatment with LPS+β-(1→3)-D-glucan (Glucan) for 3 h and 6 h. Footnote: Negative control (PBS) and positive control (LPS; 1 µg/mL). β-(1→3)-D-glucan was added at concentrations of 25, 50, and 100 µg/mL. Statistical analyses were performed by means of one-way analysis of variance (ANOVA) followed by Bonferronis' test, selected pairs. The results represent the mean ± SD of duplicate cultures of three representative experiments. *p<0.05; **p<0.01; ***p<0.001 versus positive control. ^#^p<0.05; ^##^p<0.01 versus different concentrations of glucan.

The purified β-(1→3)-D-glucan (SD-PK5) was firstly tested about its cytotoxicity and none negative effect was observed on macrophages (Figure S1). Then, the β-(1→3)-D-glucan was added to the cells and incubated for 3 h and 6 h. There was no stimulation of pro-inflammatory gene expression (data not shown). However, when the β-(1→3)-D-glucan was added concomitantly with LPS (1 µg/mL), and incubated for 3 h, it was observed an inhibition of the mRNA expression of 75.5±0.37% (IL-1β), 81.0±2.12% (TNF-α), and 81.6±0.56% (COX-2) at a concentration of 50 µg/mL (Figure 6). This inhibition was higher than the aqueous extracts, and similar to the alkaline extract (Table 2), which suggests that the isolated β-(1→3)-D-glucan is the most potent anti-inflammatory compound present in the polysaccharide extracts of *C. militaris.*

Researchers have observed that pattern recognition receptors (PRRs) of immune cells, such as dectin-1, complement receptor 3 (CR3), scavenger receptors, lactosylceramide (LacCer), and toll-like receptors (TLRs), recognize the β-glucans and initiate immune responses [15], [16]. It was demonstrated that heteropolysaccharides from *Polyporus umbellatus* and *Cordyceps militaris* activated macrophages and dendritic cells, respectively, via TLR-4 signaling pathways [46], [47]. Besides, it is well known that TLR-4 is the primary signal transducer for LPS, while TLR-2 is either a low affinity receptor for this bacterial endotoxin [48]. Taking this information into account, it is possible that the β-(1→3)-D-glucan is competing with LPS for binding the TLR-4, thus inhibiting the LPS-stimulation of pro-inflammatory genes, after 3 h of incubation. However, in the present study, the inhibitory effect of the β-(1→3)-D-glucan did not last for 6 h of incubation, which may be explained by the LPS-stimulation via TLR-2. This could be observed also for the K5 treatment (Figure 5), which is consistent because the main component of K5 is the β-(1→3)-D-glucan. The molecular mechanisms of LPS-induced macrophage activation and desensitization have been extensively investigated. These studies suggested the involvement of various kinases and that the cooperation of MyD88-dependent and MyD88-independent (TRIF-dependent) signaling is required [49]. The inhibition of any phase of these pathways leads to a negative effect of the LPS-stimulation, with no expression of pro-inflammatory genes [48], [49].

Most of the β-(1→3),(1→6)-D-glucans isolated from mushrooms are able to activate macrophages and stimulate the production of pro-inflammatory cytokines *in vitro* [15], although these effects were not observed on this study. One possible explanation could be that the activation of macrophages requires a branched polysaccharide structure. However, the linear chain of the β-(1→3)-D-glucan of *C. militaris* showed to be more efficient in inhibiting inflammation of THP-1 cells *in vitro*. Up to now, no conclusions were found in the literature pointing to the most potent β-D-glucan structure or which membrane receptor is the preferable to bind these molecules [40]. Further investigation is required to explain the anti-inflammatory effect exhibited by the β-(1→3)-D-glucan *in vitro*.

### Evaluation of the antinociceptive and anti-inflammatory properties *in vivo*

As the β-(1→3)-D-glucan of *C. militaris* showed *in vitro* anti-inflammatory activity, we decided to confirm its biological action *in vivo.* It is well known that formalin administration causes local tissue injury to the paw of mice and has been used as a model for tonic pain and localized inflammatory pain [50]. This model is constituted by two distinct phases: the neurogenic pain and the inflammatory pain. The early phase (neurogenic pain) results from the direct irritating effect on nociceptors activating primary afferent fibers, and the late phase (inflammatory pain) is related to the release of pro-inflammatory mediators, such as bradykinin, prostaglandins, and cytokines [50], [51].

**Figure 7:**
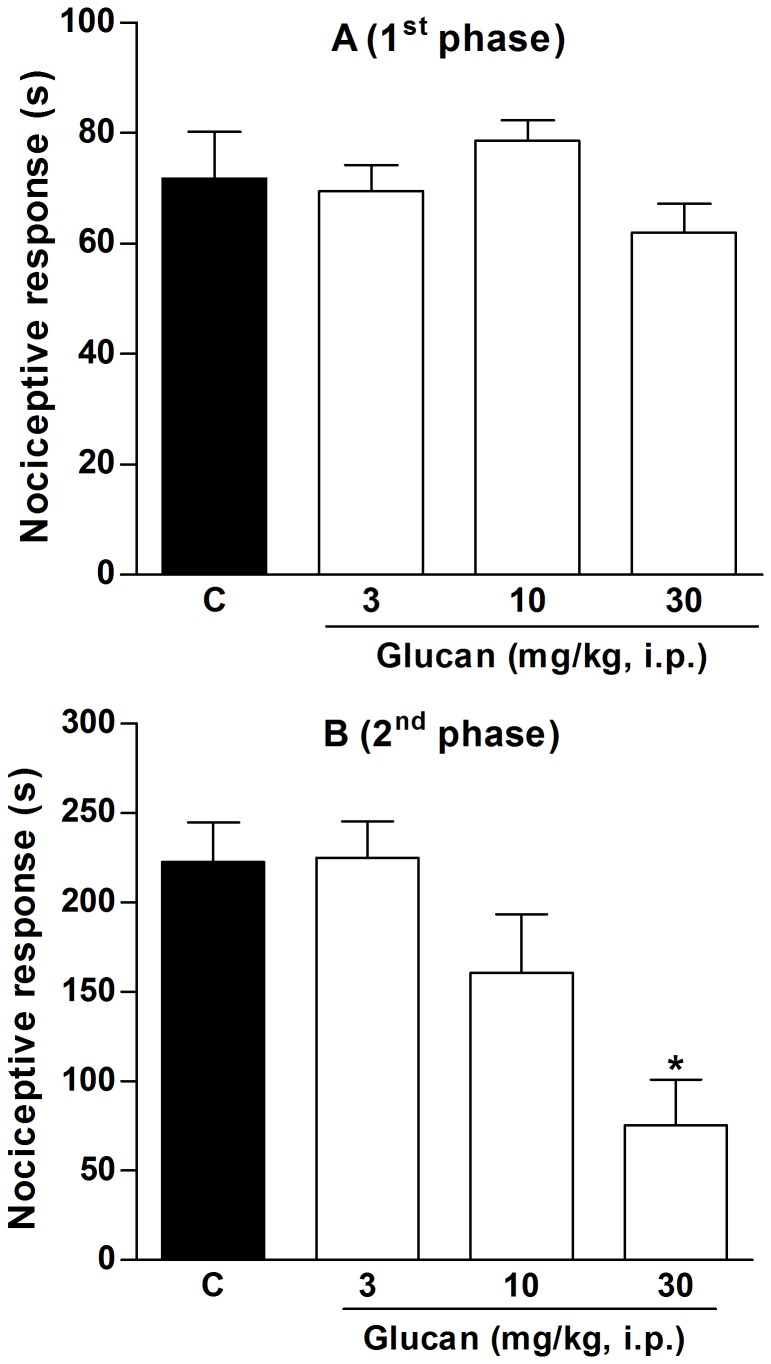
Effect of β-(1→3)-D-glucan on neurogenic (A) and inflammatory phase (B) of nociception induced by formalin in mice. Footnote: Mice received vehicle (saline plus 5% Me_2_SO, 10 mL/kg, i.p.) or β-(1→3)-D-glucan (3, 10 and 30 mg/kg, i.p.) 30 min before formalin administration. Statistical analyses were performed by means of one-way analysis of variance (ANOVA) followed by Newman–Keuls’ test. The results represent the mean ± SEM of 10–12 animals. *p<0.05 versus control group.

The results depicted in Figures 7A and 7B show that intraperitoneal administration of β-D-glucan did not inhibit the nociception on the early phase, although it significantly inhibited the nociceptive response on the inflammatory phase by 69±11%, at a dose of 30 mg/kg, when compared to control group (C: 242.5±25.4 s). Despite of this test is mainly considered a pain model, anti-inflammatory drugs can also be effective on the late phase. Indeed, it was demonstrated that classical non-steroidal anti-inflammatory drugs (NSAIDs), such as acetylsalicylic acid, indomethacin, paracetamol, and diclofenac can only attenuate the inflammatory phase of formalin-induced nociceptive response [50], [52], [53]. Thus, our results suggest that β-D-glucan isolated from *C. militaris* was effective against the inflammatory pain, with a similar anti-inflammatory effect of NSAIDs.

**Figure 8:**
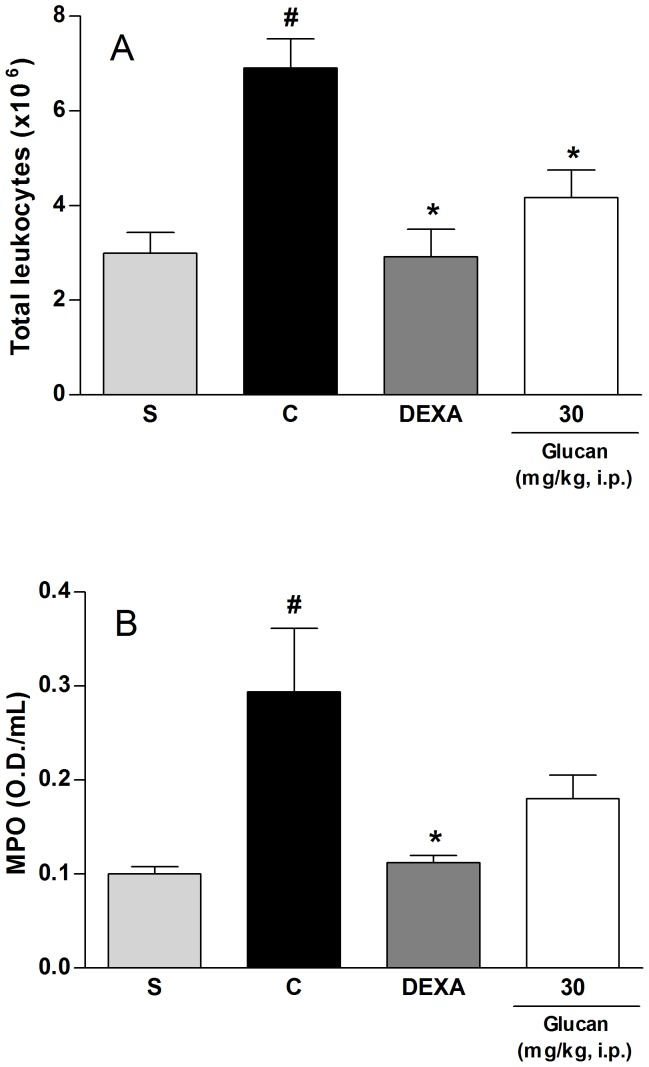
Effect of β-(1→3)-D-glucan on number of total leukocytes (A) and myeloperoxidase levels (B) induced by LPS in mice. Footnote: Mice received vehicle (saline plus 5% Me_2_SO, 10 mL/kg, i.p.), dexamethasone (DEXA, 0.5 mg/kg, i.p.), or β-(1→3)-D-glucan (30 mg/kg, i.p.) 30 min before LPS administration. Statistical analyses were performed by means of one-way analysis of variance (ANOVA) followed by Newman–Keuls’ test. The results represent the mean ± SEM of 6–8 animals. ^#^p<0.05 versus saline group; *p<0.05 versus control group.

Then, to confirm the *in vivo* anti-inflammatory activity of β-D-glucan, we performed an acute inflammation model, the peritonitis induced by LPS in mice. Intraperitoneal administration of LPS initiates an inflammatory response with recruitment and activation of leukocytes (both mononuclear cells and neutrophils), and subsequent release of pro-inflammatory mediators [54]. In the present study, we observed that intraperitoneal administration of LPS (2 µg/kg) produced an increase of leukocyte migration when compared to animals treated only with saline (S: 2.99±0.44×10^6^ cells) (Figure 8A). However, the treatment of mice with β-D-glucan, at dose of 30 mg/kg, inhibited the migration of total leukocytes to the peritoneal cavity by 70±15% compared to control group (C: 6.90±0.62×10^6^ cells). Dexamethasone, a positive control of the test, also reduced the number of total leukocytes by 100% (Figure 8A). Additionally, we also evaluated the MPO levels, an indirect marker of neutrophils, and it was observed that β-D-glucan (30 mg/kg, i.p.) did not alter the MPO levels but dexamethasone reduced them by 94±4% compared do control group (C: 0.29±0.07 O.D./mL) (Figure 8B). These results confirm the anti-inflammatory activity of β-D-glucan, reducing the migration of total leukocytes but indirectly indicating no alteration of neutrophils recruitment.

Some authors have showed that cordycepin, which is easily extracted with ethanol from *Cordyceps* species, is partly responsible for the anti-inflammatory effects of these mushrooms [23]. Although, our results demonstrated that other compounds, as the β-(1→3)-D-glucan can also exhibit such effect. The necessity of finding anti-inflammatory drugs with less side effects still remains unsolved [53], and the possibility of using a natural product as inhibitor of inflammation open a new research field based on medicinal usage of mushrooms and their compounds.

## Conclusions

The medicinal mushroom *C. militaris* was evaluated for its anti-inflammatory properties. The polysaccharide extracts from this mushroom exhibited different effects related to their monosaccharide composition. The alkaline extract, from which a linear β-(1→3)-D-glucan was isolated, showed the higher anti-inflammatory effect by the inhibition of IL-1β, TNF-α, and COX-2 expression. The β-(1→3)-D-glucan showed the same effect as well, indicating that this polymer is the most potent anti-inflammatory compound present in the polysaccharide extracts of *C. militaris.* In addition, we also observed that the isolated β-(1→3)-D-glucan presented antinociceptive and anti-inflammatory activities against formalin-induced nociception and LPS-induced peritonitis in mice.

## Supporting Information

Figure S1 **Viability of THP-1 macrophages after incubation with the extracts (CW, HW, K5) or the β-D-glucan (Glucan), for 24 h and 48 h. Footnote:** The cells were treated with 10, 50 or 250 µg/mL of extracts or glucan. Saponin was added to C+ to lysate the cells. C- received only PBS and it was set as 1.0 (100% of viable cells).
(TIF)
