# Supplementary figures and images for: Anti-Inflammatory Properties of the Medicinal Mushroom Cordyceps militaris Might Be Related to Its Linear (1→3)-β-D-Glucan
Source: PLoS One. 2014 Oct 17;9(10):e110266. doi: 10.1371/journal.pone.0110266 (PMC4201515; doi:10.1371/journal.pone.0110266)

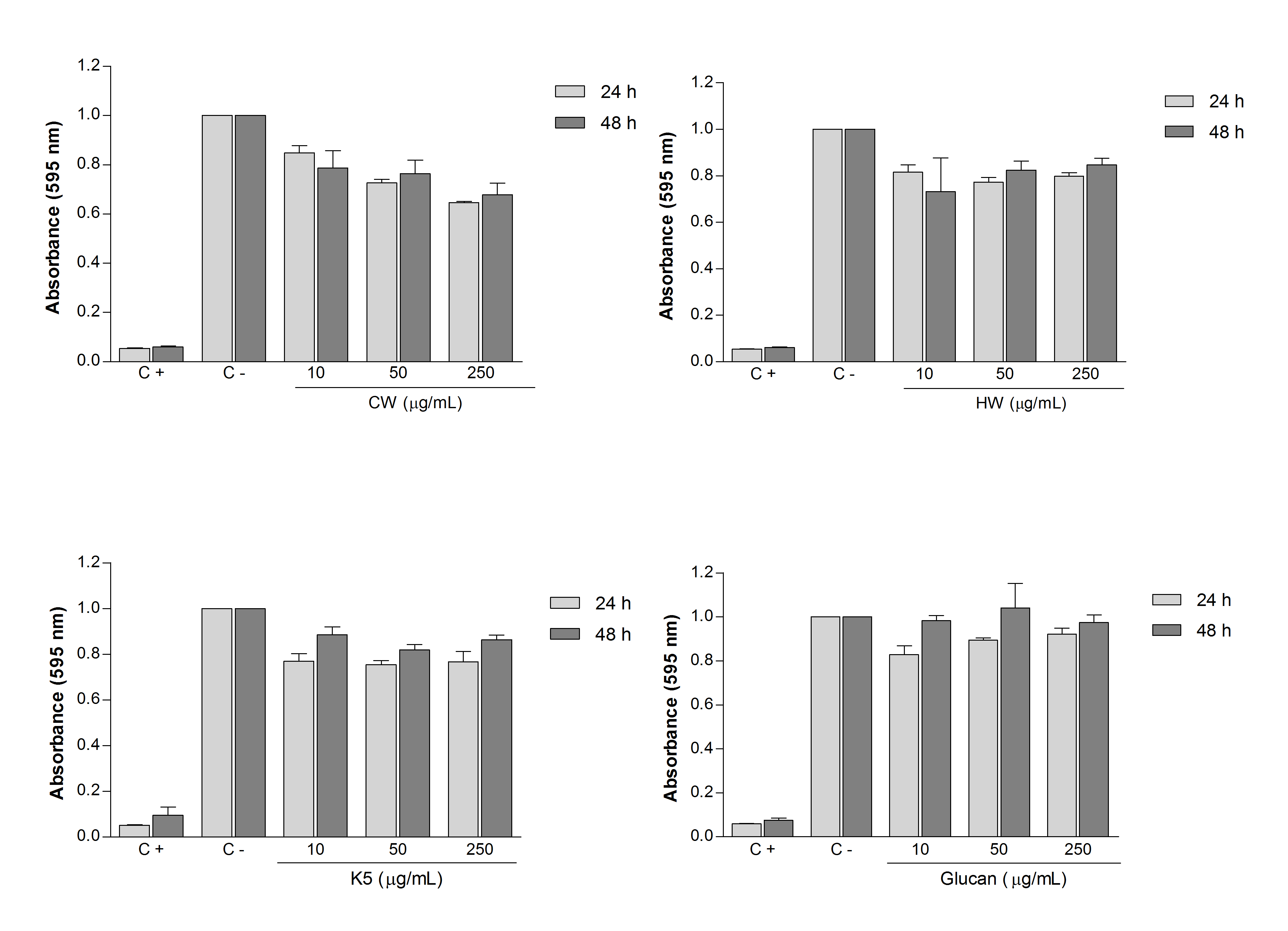

Supplement: Figure S1 — Viability of THP-1 macrophages after incubation with the extracts (CW, HW, K5) or the β-D-glucan (Glucan), for 24 h and 48 h. Footnote: The cells were treated with 10, 50 or 250 µg/mL of extracts or glucan. Saponin was added to C+ to lysate the cells. C- received only PBS and it was set as 1.0 (100% of viable cells). (TIF) [file pone.0110266.s001.tif]
